# Diseases of the Joints

**Published:** 1828-04

**Authors:** 


### HOSPITAL REPORTS,

*(Principally condensed from various Periodical Publications.)*

---

### DISEASES OF THE JOINTS.

CASE I.—*Rheumatic Inflammation, terminating in Ulceration of the Cartilages.*

SARAH HOLDER, ætatis twenty-two, was admitted into ST. GEORGE'S HOSPITAL, July 26th, 1827, under the care of Mr. BRODIE, with swelling extending from the upper part of the right thigh over the knee, for some little distance down the leg. This swelling was by no means circumscribed, but its principal seat was evidently in the lower part of the thigh and knee, above and below which it gradually passed away. It was tense, elastic, exquisitely painful on pressure, and of a glossy white, looking as bloodless as the limb of a statue. She could not bear any motion of the knee, but still the pain excited did not appear to be so much in the joint itself, as in the parts around and about it. The pain and swelling had attacked her suddenly the day before, without any precursory shivering or feverishness whatever. She had received no injury to her knowledge, nor had she been particularly exposed at the time to wet or cold. She had, however, been subject, for a month or more, to rheumatic pains in the elbows and shoulders, and was leading a life of prostitution.

The skin was hot and dry; tongue coated, and brownish in the centre; pulse eighty-four; bowels confined; countenance expressive of much suffering.

The local treatment consisted of an alternation of leeches and blisters; the constitutional, at first of saline purges with antimony. and, after a few days, the exhibition of calomel and opium, until the mouth became affected. Under this plan the pain was considerably relieved, but never entirely dissipated, whilst the swelling of the upper part of the thigh and leg almost wholly subsided.

In this state she was removed, with the other patients, to the opposite side of the house, in consequence of some arrangements connected with the new hospital, soon after which she was seized with fever. The swelling of the knee and parts around increased, and the pain was greatly aggravated. There were hectic symptoms, and the constitution now began to suffer. She was kept gently under the influence of mercury, and a succession of blisters was applied to the knee.

On the 6th of September, the calomel was omitted, in consequence of violent purging. Pil. Saponis cum Opio, blisters, leeches, Belladonna fomentations, Belladonna plasters, and lastly a caustic issue to the knee, were employed, sometimes with temporary, but never with any permanent relief. The disease in the joint went on from bad to worse, and the sufferings of the patient were so excessive that her screams could be heard from one end of the ward to the other. An ulcer formed over the sacrum, and somewhat relieved her; for, in proportion as this got worse, the pain in the knee got better.

On the 15th October, bilious vomiting and diarrhœa, with great tenderness of the abdomen, came on; and, at seven P.M. on the 16th she died, worn out with suffering.

*Dissection.*—On cutting into the knee-joint, not a particle of purulent matter was found in its cavity, but the cartilages of the condyles of the femur (particularly the inner one) of the head of the tibia, and of the patella, were in a state of ulceration, exposing the cancellated structure of the bone beneath. An incision was made into the thigh, but its tissues were unaltered, there being no thickening whatever of the periosteum.

CASE II.—Sarah Mooley, æt. thirty-seven, was admitted into St. George's Hospital, December 12th, 1827.

On the 1st of the month she was seized with pain in the left knee, which swelled a good deal in the course of that night. During the whole of next day she suffered considerably, and in the evening was attacked with fever. She now applied a poultice and fomentation, and on the 4th was seen by the parish surgeon, who ordered eight leeches to the knee, and gave her some medicine. At the time of her admission, there was the same train of symptoms and appearances as have been described in the preceding case: there was the same diffused swelling, still however concentrated about the knee; the same want of œdema; the same exquisite pain on pressure of any part, or motion in any way. The limb, as in the former instance, had that peculiar opaque and marbled whiteness, which made it look as if it belonged to a statue rather than a living person, but the integument was neither so glossy nor so tense. There was great pain, independent of pressure, shooting from the knee down to the toes, and up to the groin; it was not aggravated at night; it was worse when the limb was hot.

The patient was a washerwoman, and of an extremely delicate, scrofulous aspect; appetite bad, pulse quick and irritable, countenance flushed and anxious. Leeches, lotions, &c. were employed; and on the 17th she was ordered Calomel and Opium, with Colchicum in Camphor mixture.

The limb now became more inclined to be œdematous; and, on the 18th, the swelling of the thigh was perceptibly diminished, whilst the leg was swollen to the instep, and distinctly œdematous. Tongue dry and chapped; mouth parched; pulse quick and hard; much anxiety of countenance.

On the 21st, the Calomel and Opium were omitted, and she was ordered

Hirudines xij. genu.—Cal. gr. ss.; Pulv. Ip. comp. gr. v. sextâ quâque horâ.—Inungatur fem. Lin. Ammon. fortius.

The leeches were repeated on the 24th, 28th, and 31st; and a lotion, containing two drachms of the Tinct. Belladon. to the pint, kept applied to the limb.

14th.—Leeches have been again applied, and she has been taking the Haust. Salin. with Vin. Ant. and Magn. Sulph. for the last week. In consequence of excessive nausea, the medicine is to be omitted. She is suffering excessively this afternoon from the pain, which shoots from the knee up and down the limb. She gets little rest at nights, but has no very decided hectic symptoms.

---

### *Disease of the Elbow-Joint, with Removal of Part of the Bones.*

A GIRL, nineteen years of age, bearing the scars of an old scrofulous affection upon the back of the left hand, was admitted at LA CHARITÉ with a white swelling of the elbow-joint, with great enlargement of the soft parts, caries of the extremities of the bones, and several fistulous openings giving vent to a sanious discharge. The constitution of the patient had not suffered in any great degree; nevertheless it was evident that the disease could only be got rid of by amputation of the arm, or removal of the diseased portions of bone. M. Roux preferred the latter, and the operation was performed December 9th.

The patient being placed upon her belly, and the diseased arm brought from the trunk: two incisions, two inches in length, were made along the external and internal edge of the humerus parallel to the axis of the limb; these incisions reached as low as the joint. A transverse incision across the posterior part of the articulation joined the two former, and from these three incisions a quadrilateral flap resulted, which was detached from below upwards. The humerus being thus exposed, was isolated, by means of the knife, from the soft parts to which it was attached; a thin piece of wood was inserted between these parts and the bone, which was then sawn off just above its tuberosities. The lateral incisions were then prolonged below upon the edges of the forearm, for the space of two or three fingers' breadth; a flap similar to the first was detached from above downwards; the upper extremity of the radius was separated from the soft parts and from the ulna, and the radius was removed above its tuberosity. The extremity of the ulna was then isolated, preserving the insertion of the brachialis anterior, and the extremity of the bone was sawn off immediately below its articular surfaces: by this contrivance the lower insertions of the brachialis and biceps were preserved. The median and radial nerves included in the mass of the soft parts anteriorly were not touched, but the cubital nerve was cut, and even lost a portion of its substance. The wound produced by this operation was large, deep, and formed in indurated and diseased tissues. The edges were brought together, and maintained by fifteen points of interrupted suture; lint spread with cerate was applied upon the wound, and fixed there by compresses and a bandage. The limb was placed in a hollow splint of tin.

On the day of the operation, the patient experienced violent pain, and could not sleep on the following night. On the 10th, there was pain in the epigastrium, increased on pressure; intense headache; violent thirst; white, moist tongue; pulse moderately frequent, but hard.

Low diet and diluents were ordered.

12th, (fourth day after the operation.)—The wound was dressed. The bandages were scarcely moistened with a bloody and purulent serum; very little tumefaction of the wound. The sutures were removed, and the edges appeared united; the incisions were marked by straight lines of a red colour.

Simple dressing.—Jalap, with two drachms of Syrup of Poppies, in the evening.

Suppuration soon became more abundant, but bore no proportion to the apparent extent of the wound; the radial and cubital incisions were separated more considerably, the one at its upper, the other at its lower extremity, and discovered the opening of a sinus, which evidently led into the cavity resulting from the removal of the bones. Excepting this, every thing improved from day to day: the appetite revived, the sleep was good, and the patient recovered her spirits. After employing sticking plaster for some days, simple cerate was employed, and two pasteboard splints supplied the place of the tin case.

January 10th.—The suppuration is good, but its quantity has not much diminished; a portion of it still comes from the bottom of the wound. The transverse incision is in a great measure healed; the extremities of the bones of the forearm and arm do not meet; the cubital edge of the ring finger has regained its feeling, but the little finger may be pinched without the patient's being at all conscious of it. The patient's health is perfectly good.

This is the third operation of the kind M. Roux has performed, and they have all succeeded. One of the former patients died afterwards of phthisis; the other is still alive. No callus is formed at the extremities of the bones, they seem to be replaced by flexible fibro-cellular tissue: the forearm is moveable; it cannot be, indeed, bent or straightened at pleasure, but the patient gives it these motions with the assistance of the other hand. This man is a grinder by trade, and finds the limb very useful.

---

